# Supplementary material for: Transcriptomic Insights into Mechanisms of Early Seed Maturation in the Garden Pea (Pisum sativum L.)
Source: Cells. 2020 Mar 23;9(3):779. doi: 10.3390/cells9030779 (PMC7140803; doi:10.3390/cells9030779)

Sprint−2(10) vs Zhongw

Global Summary

%DE = 0.89

genes with fdr < 0.2 = 536 (284 + / 252 −)

genes with fdr < 0.1 = 484 (251 + / 233 −)

genes with fdr < 0.05 = 481 (249 + / 232 −)

genes with fdr < 0.01 = 362 (182 + / 180 −)

genes in genesets = 0

<FC> = 0

<t−score> = −123.03

<p−value> = 0

<fdr> = 0.11

Portrait

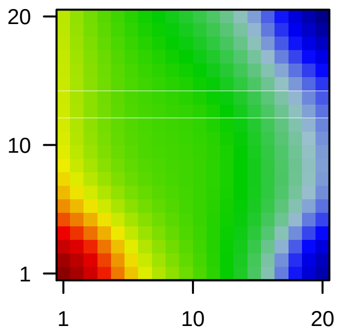

Top 100 DE genes

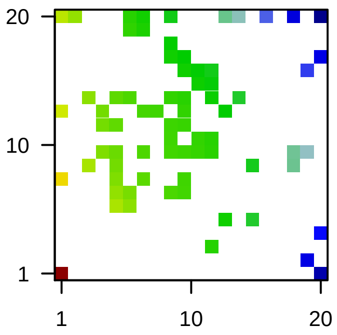

Global Genelist

Rank	ID	log(FC)	p−value	fdr	Metagene	Description
1	TRINITY_DN1	−11.1	7e−15	7e−10	20 x 20	
2	TRINITY_DN1	−19.01	1e−11	4e−09	20 x 1	
3	TRINITY_DN1	−0.01	8e−11	4e−09	9 x 12	
4	TRINITY_DN4	5.77	1e−10	4e−09	1 x 1	
5	TRINITY_DN2	−5.88	2e−10	4e−09	20 x 1	
6	TRINITY_DN6	−1.86	2e−10	6e−07	20 x 1	
7	comp40744_c	−0.07	9e−09	1e−06	13 x 13	
8	TRINITY_DN2	0	5e−08	1e−06	9 x 11	
9	TRINITY_DN7	−0.15	6e−08	1e−06	15 x 5	
10	TRINITY_DN1	2.16	7e−08	1e−06	1 x 1	
11	TRINITY_DN4	−2.16	9e−08	1e−06	18 x 20	
12	comp4770_c0	0.02	1e−07	1e−06	7 x 13	
13	TRINITY_DN1	−0.11	1e−07	2e−06	12 x 15	
14	TRINITY_DN1	0.09	2e−07	2e−06	4 x 12	
15	TRINITY_DN3	−2.27	2e−07	2e−06	20 x 1	
16	TRINITY_DN8	0.08	2e−07	2e−06	6 x 7	
17	TRINITY_DN1	−0.06	2e−07	2e−06	7 x 20	
18	TRINITY_DN6	1.81	2e−07	2e−06	1 x 1	
19	TRINITY_DN1	0	3e−07	2e−06	11 x 10	
20	TRINITY_DN1	−0.06	3e−07	2e−06	12 x 11	

p−values

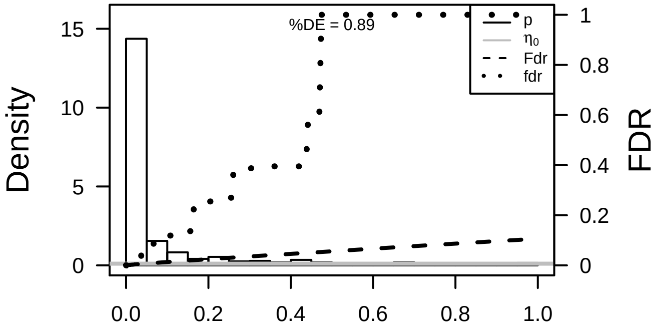

Supplement: Supplementary file 1 [file cells-09-00779-s001.zip › Supplementary materials/FileS3.zip!/FileS2/Hormones - Results/Summary Sheets - Differences/Reports/Sprint.2.10..vs.Zhongwan.6.25..pdf]
